# Deciphering the Origin of DNA Viruses (Replication-Associated Parvo-NS1) That Infect Vertebrates from Invertebrate-Infecting Viruses

**DOI:** 10.1128/spectrum.04570-22

**Published:** 2023-06-22

**Authors:** Perumal Arumugam Desingu, T. P. Rubeni, Nagalingam R. Sundaresan

**Affiliations:** a Department of Microbiology and Cell Biology, Indian Institute of Science, Bengaluru, India; Oklahoma State University College of Veterinary Medicine

**Keywords:** origin of parvovirus, origin of adenovirus, origin of herpesvirus, origin of papillomavirus, origin of polyomavirus

## Abstract

DNA replication is a standard and essential function among DNA viruses; however, this functional domain's common ancestor, origin, and evolutionary path in invertebrate- and vertebrate-infecting viruses are not yet fully understood. Here, we present evidence, using a combination of phylogenetic relationships, coevolution, and CLANS (cluster analysis of sequences) analysis, that the parvo-NS1 domain (nonstructural protein NS1, DNA helicase domain) of these DNA viruses that infect vertebrates potentially originated from the invertebrate (Platyhelminthes) parvo-NS1 domain of parvovirus-related sequences (PRSs). Our results suggest that papillomaviruses and the parvovirus subfamilies *Densovirinae* and *Hamaparvovirinae* DNA helicase evolved directly from the Platyhelminthes NS1 domain (PRSs). Similarly, the parvovirus subfamily *Parvovirinae* NS1 domain displayed evolutionary heritage from the PRSs through *Hamaparvovirinae.* Further, our analysis also clarified that herpesviruses and adenoviruses independently obtained the parvo-NS1 domain from *Dependoparvovirus* (*Parvovirinae*). Furthermore, virus-host coevolution analysis revealed that the parvovirus NS1 domain has coevolved with hosts, from flatworms to humans, and it appears that the papillomavirus may have obtained the DNA helicase during the early stages of parvovirus evolution and later led to the development of the DNA helicase of adomavirus and polyomavirus. Finally, herpesviruses and adenoviruses likely inherited the parvo-NS1 domain from *Dependoparvovirus* in the later stages of evolution. To the best of our knowledge, this is the first evolutionary evidence to suggest that the DNA helicase of viruses that infect vertebrates originated from the invertebrate PRSs.

**IMPORTANCE** DNA replication of DNA viruses is an essential function. This allows DNA replication of viruses to form virus particles. The DNA helicase domain is responsible for this primary function. This domain is present in parvoviruses, papillomaviruses, polyomaviruses, herpesviruses, and adenoviruses. But little is known about the common ancestor, origin, and evolutionary path of DNA helicase in invertebrate- and vertebrate-infecting viruses. Here, we report the possibility of the origin of DNA viruses (DNA helicase) infecting vertebrates from Platyhelminthes (invertebrate) PRSs. Our study established that the parvovirus subfamily *Parvovirinae* NS1 domain displayed evolutionary heritage from the Platyhelminthes PRSs through *Hamaparvovirinae*. Furthermore, our study suggests that the papillomavirus DNA helicase may have evolved in the early stages of parvovirus evolution and then led to the development of the adomavirus and polyomavirus. Our study suggests that the herpesviruses and adenoviruses likely inherited the parvo-NS1 domain through gene capture from *Dependoparvovirus* in the later stages of parvovirus evolution in their hosts.

## INTRODUCTION

DNA viruses contribute to different viral infections in animals and humans. Among DNA viruses, parvoviruses are linear single-stranded DNA viruses that infect hosts from invertebrates to vertebrates. Recently, parvoviruses have been classified into three subfamilies: *Parvovirinae*, *Densovirinae*, and *Hamaparvovirinae*, affecting vertebrates, invertebrates, and both vertebrates and invertebrates, respectively (1). The subfamily *Hamaparvovirinae* is divided into the genera *Hepanhamaparvovirus* (*Decapod hepanhamaparvovirus 1*), *Penstylhamaparvovirus* (*Decapod penstylhamaparvovirus 1*), *Brevihamaparvovirus* (*Dipteran brevihamaparvovirus 1* and *2*), *Ichthamaparvovirus*, and *Chaphamaparvovirus*, with the first three genera affecting invertebrates and the last two genera affecting aquatic and terrestrial animals, respectively (1–3). Furthermore, dependoparvoviruses in *Parvovirinae* depend on helper viruses, such as adenoviruses or herpesviruses, to replicate their genome (4, 5). Recently, parvovirus-related sequences (PRSs) in invertebrates, including platyhelminths and arthropods, have been well-documented but have not yet been officially classified in any genus (6, 7). Parvoviruses encode nonstructural (NS) proteins and structural proteins (VPs). Among these proteins, NS1 is a multifunctional protein required for viral DNA replication and has a conserved helicase superfamily 3 (SF3) domain (parvo-NS1 domain/DNA helicase domain) (1). The strong monophyletic lineage at the complete NS1 protein and SF3 helicase domain level has led to the classification of parvoviruses at the subfamily and family levels, respectively (1).

Next, the DNA virus families *Papillomaviridae* and *Polyomaviridae* are nonenveloped viruses with circular double-stranded DNA (dsDNA) genomes and are well known to infect vertebrates (8–10). Using specific sequences discovered as early as 30 years ago, the DNA helicase of papillomavirus E1 and the large T-antigen polyomavirus have been shown to have sequence similarity with the parvovirus replication protein (NS1) (11–13). Among the proteins present in adenoviruses, the replication gene (SF3 helicase domains) has been found to have homologs with those of polyomaviruses and papillomaviruses, and the capsid proteins with the major and minor capsid proteins of adenoviruses (14). In contrast, only members of the *Betaherpesvirinae* subfamily human herpesvirus 6 (HHV-6), rat cytomegalovirus (RCMV), and bat Miniopterus schreibersii betaherpesvirus (MsHV) of the *Herpesviridae* family encode the U94/Rep domain homolog to the parvovirus NS1 domain (15–18). Similarly, only aviadenoviruses belonging to the genus *Aviadenovirus* of the *Adenoviridae* family encode ORF2, a homolog of the NS1 domain of parvoviruses (19). Overall, DNA viruses, such as parvoviruses, adomaviruses, papillomaviruses, and polyomaviruses, require the parvo-NS1 homologous DNA helicase domain to initiate viral DNA replication. Moreover, some herpesviruses and aviadenoviruses that contain the parvo-NS1 protein in their genome may or may not require the parvo-NS1 homologous DNA helicase domain to initiate viral DNA replication to a large extent. However, parvo-NS1, primarily required for the replication of other DNA viruses, is also present in some herpesviruses and adenoviruses, and the evolutionary origin and evolutionary path of parvo-NS1 in these viruses are not yet fully understood.

The answers to the above questions require a large-scale sequence-based evolutionary analysis. Good-quality multiple-sequence alignments play an essential role in accurately determining the phylogenetic relationship between organisms. However, the ambiguously aligned regions that exist when examining this DNA helicase in such diverse DNA virus families can also affect the exact nature of the phylogenetic relationship analyzed. BMGE (block mapping and catering with entropy), Gblocks, Noisy, or trimAl methods can be used to trim sequences with vaguely aligned areas to improve the accuracy of the phylogenetic relationship (20). When examining genes in such diverse DNA virus families, the number of possible connections is likely to be large, significantly affecting the accuracy of the alignment-based phylogenetic relationship. CLANS-based (cluster analysis of sequences) pairwise sequence similarity analyses help determine the exact evolution of proteins beyond these effects (21). The evolutionary origin of the replication gene of some parvovirus, papillomavirus, and polyomavirus sequences has been shown through CLANS analysis (22) and characterization of circular DNA viruses (23, 24). However, it is not yet fully understood whether this homologous DNA helicase in diverse DNA virus families originated from common ancestors, what these common ancestors are, or whether these came about from convergent evolution or gene capture, and the evolutionary path of parvo-NS1 in the parvoviruses, invertebrate-infecting DNA viruses, and vertebrate-infecting DNA viruses are not fully understood. Hence, we are interested in finding the evolutionary origin of parvo-NS1/SF3 helicases within the *Parvoviridae* family of viruses infecting invertebrates and vertebrates and among other DNA viruses infecting vertebrates. Further, if parvoviruses coevolved with their respective hosts, they would help determine the path of evolution in the DNA virus families with this homologous DNA helicase.

In the current study, we systematically explored the path of DNA helicase evolution across viral families using more than 3,000 sequences of parvo-NS1 and parvo-NS1 domains (DNA helicase domain) from parvoviruses, papillomaviruses, adomaviruses, polyomaviruses, herpesviruses, and aviadenoviruses using phylogenetic tree-based MAFFT alignment, MAFFT, and BMGE curation-based, CLANS, and virus-host coevolution. Our analysis showed that the papillomavirus DNA helicase evolved from the Platyhelminthes PRS early in the coevolution between parvoviruses and their hosts, leading to the development of polyomaviruses. We then further clarified the evolution of herpesvirus and adenovirus DNA helicases from dependoviruses in the later stages of the coevolution of parvoviruses and their respective hosts. Overall, our results shed light on the potential evolutionary origin of the DNA helicase of DNA viruses that infect vertebrates from the common ancestor of invertebrate PRSs.

## RESULTS

### Phylogenetic relationship of parvo-NS1 with other DNA viruses.

**FIG 1:**
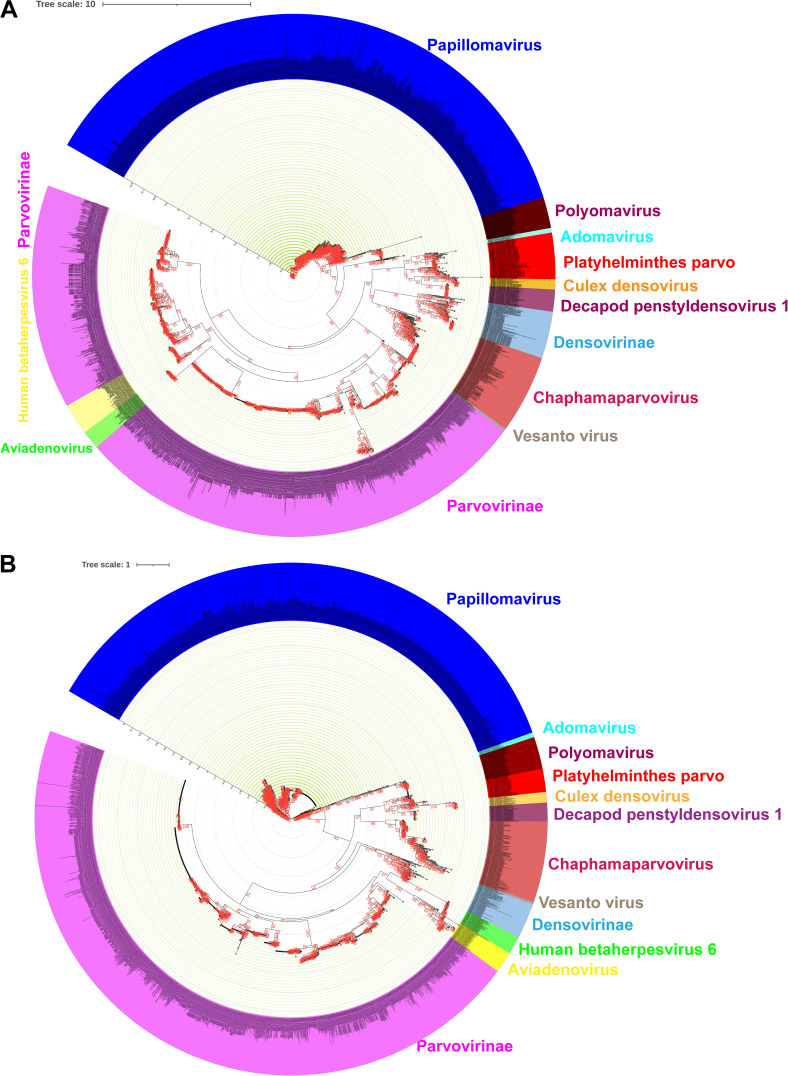
Phylogenetic relationship of parvo-NS1 complete protein and parvo-NS1 domains with other DNA viruses. (A) The phylogenetic tree depicts parvoviruses parvo-NS1's entire protein-based phylogenetic relationship with other DNA viruses. A total of 3,152 amino acid sequences, such as the complete protein of parvovirus parvo-NS1 and related proteins in other DNA viruses, were used in this analysis (the details of strains that belong to different clusters are presented in Data Set S1 in the supplemental material). (B) The phylogenetic tree depicts the parvo-NS1 domain-based phylogenetic relationship of parvoviruses with other DNA viruses. A total of 2,974 amino acid sequences, such as the parvovirus parvo-NS1 domain and its related sequences in other DNA viruses, were used in this analysis (the details of strains that belong to different clusters are presented in Data Set S2 in the supplemental material).

To explore the evolution of parvo-NS1 across DNA viruses, we retrieved 3,152 amino acid sequences related to parvo-NS1 from parvoviruses, papillomaviruses, adomaviruses, polyomaviruses, herpesviruses, and adenoviruses from the NCBI public database (the details of strains that belong to different clusters are presented in Data Set S1 in the supplemental material). We then performed a phylogenetic analysis of the full-length protein sequence of parvo-NS1 and the protein sequences of parvo-NS1-related papillomavirus E1, adomaviruses parvo-NS1, polyomavirus large T-antigen, aviadenovirus ORF2, and betaherpesvirus U94/Rep. Phylogenetic analysis revealed a relationship based on the amino acid sequence of parvo-NS1, papillomavirus E1, adomaviruses parvo-NS1, the polyomavirus large T-antigen, aviadenovirus ORF2, and the U94/Rep of the betaherpesvirus (Fig. 1A). Furthermore, papillomaviruses, adomaviruses, and polyomaviruses formed a separate branch from the other viruses (Fig. 1A) (a similar result was obtained in the phylogenetic analysis performed by PhyML Galaxy version 3.3_1, taking only representative sequences from 3,152 amino acid sequences; data not shown). In this analysis, Platyhelminthes PRSs, *Culex* densovirus, *Decapod penstyldensovirus 1*, *Densovirinae*, and *Chaphamaparvovirus* formed a distinct clade. Similarly, *Parvovirinae* formed a distinct clade (Fig. 1A). Remarkably, human betaherpesvirus 6 and *Aviadenovirus* were grouped between *Parvovirinae* viruses (Fig. 1A). Although it is uncertain what the common ancestor is in this analysis, the clade comprising *Densovirinae*, *Culex* densovirus, *Decapod penstyldensovirus 1*, and the Platyhelminthes PRSs may have evolved into links to *Parvovirinae* and *Chaphamaparvovirus*. Although papillomaviruses, adomaviruses, and polyomaviruses have been grouped outside parvoviruses, it is not completely ruled out that they may have originated from convergent evolution, but it is not entirely possible to determine which of these viruses may have originated from parvoviruses. However, there is a good chance that these heterogeneous DNA virus families will have similarities in functional proteins required for DNA replication.

### Phylogenetic relationship of the parvo-NS1 DNA helicase domain with other DNA viruses.

The presence of the parvo-NS1 (DNA helicase/SF3) domain has been suggested as a distinct characteristic to classify the parvovirus family (1), so we explored whether there is any indication of the evolution of these DNA virus families in the DNA helicase domain range. Notably, we observed a better sequence alignment in the DNA helicase domain of parvo-NS1, papillomavirus E1, adomaviruses parvo-NS1, the polyomavirus large T-antigen, the *Aviadenovirus* ORF2, and U94/Rep of betaherpesvirus (a total of 2,974 amino acid sequences used; the details of strains that belong to different clusters are presented in Data Set S2 in the supplemental material). We then subjected the well-aligned DNA helicase domain sequences to phylogenetic analysis. Similar to the complete parvo-NS1 protein, phylogenetic analysis at the level of the parvo-NS1 DNA helicase domain revealed papillomaviruses, adomaviruses, and polyomaviruses to be an out-group from other viruses (Fig. 1B) (a similar result was obtained in the phylogenetic analysis performed by PhyML Galaxy version 3.3_1, taking only representative sequences from 2,974 amino acid sequences; data not shown). Platyhelminthes PRSs, *Culex* densovirus, *Decapod penstyldensovirus 1*, *Densovirinae*, and *Chaphamaparvovirus* formed a distinct clade, but *Parvovirinae* formed out group from these viruses (Fig. 1B). Similar to the complete parvo-NS1 protein, phylogenetic analysis at the parvo-NS1 DNA helicase domain level revealed human betaherpesvirus 6 and *Aviadenovirus* were grouped between *Parvovirinae* viruses (Fig. 1A and B). After this, since DNA helicase/SF3 motif is present in the parvo-NS1 domain amino acid sequences used in this present study, we are interested to find out what result will be obtained if the sequences used in the previous study (22) to detect the evolutionary development of SF3 domain in other viruses (circular replication [Rep]-encoding single-stranded [CRESS] DNA virus, *Circoviridae*, genomoviruses, geminiviruses, *Nanoviridae*/*Alphasatellitidae*, smacovirus, plasmid-CRESS [pCRESS] sequences) and plasmids are used as an outgroup in phylogenetic analysis. In this analysis, the parvo-NS1 domain amino acid sequences used in the present study align well with the SF3 motif (Walker A, Walker B, Motif C, and Arg finger) (22) in other viruses and plasmids in previous studies (a total of 3,647 amino acid sequences were used; the details of strains that belong to different viruses are presented in Data Set S2a). Furthermore, sequences containing the SF3 motif in other viruses and plasmids used in the previous study formed an outgroup in the phylogenetic analysis, and the topology of the parvo-NS1 domain amino acid sequences used in the present study did not change significantly (see Fig. S1 in the supplemental material). Collectively, the functional domain-based analysis also shows that these diverse DNA virus families are evolutionary, but it is somewhat difficult to predict their evolutionary path.

### MAFFT alignment and BMGE curation-based phylogenetic relationship of the parvo-NS1 domain with other DNA viruses.

**FIG 2:**
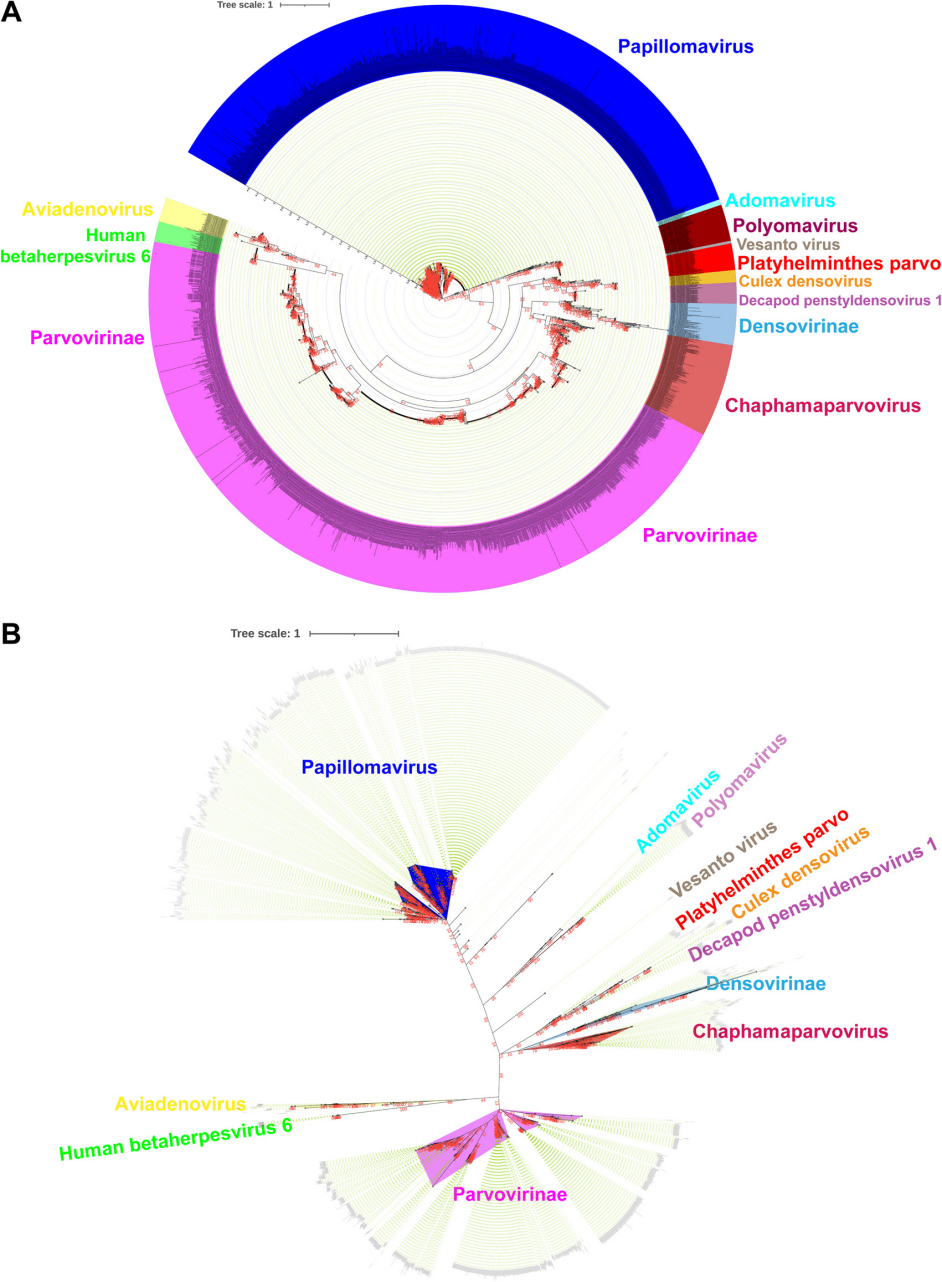
Phylogenetic relationship of parvo-NS1 domains with other DNA viruses using MAFFT alignment and BMGE curation-based phylogenetic analysis. The phylogenetic tree depicts the parvo-NS1 domain-based phylogenetic relationship of parvoviruses with other DNA viruses using MAFFT alignment and BMGE curation-based phylogenetic analysis, rooted tree (A), and unrooted tree (B). The details of strains that belong to different clusters are presented in Data Set S2.

To better understand the evolutionary origin of the diversified DNA virus DNA helicase domain, we performed a phylogenetic analysis based on MAFFT alignment and BMGE curation (a total of 2,974 amino acid sequences used; the details of strains that belong to different clusters are presented in Data Set S2). In this analysis, we also observed that viruses clustered like the complete parvo-NS1 protein (Fig. 2A) (a similar result was obtained in the phylogenetic analysis performed by PhyML Galaxy version 3.3_1, taking only representative sequences from 2,974 amino acid sequences; data not shown). Next, we performed an unrooted tree to determine the evolutionary path of these viruses. This analysis also showed that human betaherpesvirus 6 and aviadenoviruses are grouped between the *Parvovirinae* and other parvoviruses (Fig. 2B). In addition, *Parvovirinae* and papillomaviruses appeared to have two different extreme evolutions (Fig. 2B). Platyhelminthes PRSs, *Culex* densovirus, *Decapod penstyldensovirus 1*, *Densovirinae*, and *Chaphamaparvovirus* appear to be the evolutionary link between *Parvovirinae* and papillomaviruses; Platyhelminthes PRSs were more likely to appear first in the evolution (Fig. 2B). Predicting the evolutionary path is also difficult in this analysis, but it appears that these viruses are evolutionarily related.

### Pairwise sequence similarity relationship of parvo-NS1 with other DNA viruses.

To better understand the evolutionary pathway for DNA viruses and their common ancestors, we performed pairwise sequence similarity relationship-based cluster analysis (CLANS). First, we analyzed the entire length of the protein sequence of parvo-NS1 and its relative papillomavirus E1. For this analysis, we used 4,362 amino acid sequence details presented in Data Set S3 in the supplemental material. At the *P* value threshold of 1.0e−13, the parvovirus family formed four distinct clusters as follows: Platyhelminthes PRSs, *Culex* densovirus, and *Decapod penstyldensovirus 1* in one cluster and *Densovirinae*, *Chaphamaparvovirus*, and *Parvovirinae* as the three others (see Fig. S2A in the supplemental material) (the details of strains that belong to different clusters are presented in Data Set S3a). Interestingly, papillomavirus E1 revealed a direct evolutionary link with the cluster formed by Platyhelminthes PRSs, *Culex* densovirus, and *Decapod penstyldensovirus 1* (Fig. S2A). These results suggest that papillomavirus E1 may have originated from a parvovirus that infects invertebrates.

**FIG 3:**
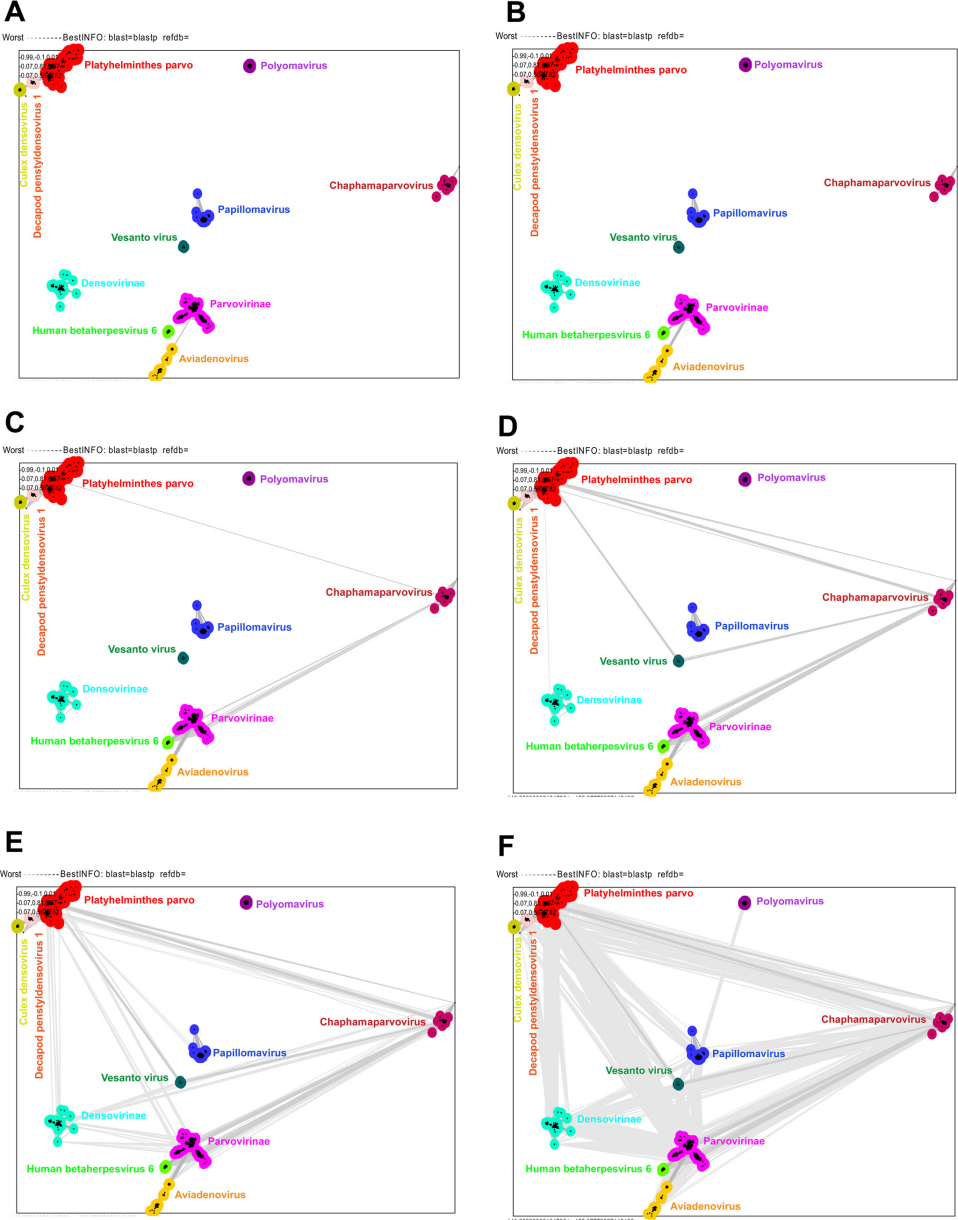
The CLANS pairwise similarity relationship of parvo-NS1 with other DNA viruses. CLANS (pairwise similarity network) analysis was carried out using a total of 3,118 amino acid sequences, such as the complete protein of parvovirus parvo-NS1 and related proteins in other DNA viruses. The details of strains that belong to different clusters are presented in Data Set S4 in the supplemental material. (A) The *P* value threshold of 1e–42 in CLANS is used to show the lines connecting the sequences. (B) The *P* value threshold of 1e–38 in CLANS is used to indicate the lines connecting the sequences. (C) The *P* value threshold of 1e–20 in CLANS is used to indicate the lines connecting the sequences. (D) The *P* value threshold of 1e–18 in CLANS is used to indicate the lines connecting the sequences. (E) The *P* value threshold of 1e–15 in CLANS is used to indicate the lines connecting the sequences. (F) The *P* value threshold of 1e–8 in CLANS is used to indicate the lines connecting the sequences.

To explore the evolutionary path of different DNA virus families in-depth, we retrieved the full-length sequences of parvo-NS1, papillomavirus E1, polyomavirus large T-antigen, *Aviadenovirus* ORF2, and the U94/Rep of betaherpesvirus from the NCBI database and performed CLANS analysis. For this, we used 3,118 amino acid sequences and provided the sequence details in Data Set S4 in the supplemental material. At a *P* value threshold of 1e−48, *Culex* densovirus, *Decapod penstyldensovirus 1*, Platyhelminthes parvoviruses, *Densovirinae* viruses, *Chaphamaparvovirus*, *Parvovirinae*, papillomavirus, polyomavirus, *Aviadenovirus*, and betaherpesvirus formed independent orphan clusters without mutual evolutionary contact (Fig. S2B) (the details of strains that belong to different clusters are presented in Data Set S4a). Next, at a *P* value threshold of 1e−44, Platyhelminthes PRSs showed an evolutionary link with *Decapod penstyldensovirus 1* (Fig. S2C). Furthermore, at a *P* value threshold of 1e−42, the Platyhelminthes PRSs demonstrated evolutionary association with *Culex* densovirus through the *Decapod penstyldensovirus 1* (Fig. 3A), and *Parvovirinae* has been shown to have an evolutionary relationship with *Aviadenovirus* (Fig. 3A). At a *P* value threshold of 1e−38, *Parvovirinae* has an evolutionary link with human betaherpesvirus 6 (Fig. 3B). Interestingly, at a *P* value threshold of 1e−20, Platyhelminthes PRSs revealed a direct relationship with *Chaphamaparvovirus* and showed an evolutionary association with *Parvovirinae* via *Chaphamaparvovirus* (Fig. 3C). Remarkably, at a *P* value threshold of 1.2e−18, Platyhelminthes PRSs established a direct evolutionary link with the *Densovirinae* viruses (Fig. 3D). Likewise, at a *P* value threshold of 1.2e−15, Platyhelminthes PRSs showed a direct evolutionary link with papillomaviruses (Fig. 3E). In addition, Platyhelminthes PRSs, *Densovirinae*, *Chaphamaparvovirus*, and *Parvovirinae* exhibited an evolutionary interconnection, at a *P* value threshold of 1.2e−15 (Fig. 3E). Aviadenovirus and human betaherpesvirus 6 are closely related to *Parvovirinae*; however, they did not show a direct evolutionary link with other viruses (Fig. 3E). Finally, at a *P* value threshold of 1.2e−8, papillomaviruses showed a direct evolutionary link with polyomaviruses (Fig. 3F). From this combination, it can be inferred that Platyhelminthes PRSs may have been a common ancestor to these DNA virus families. From the common ancestors of Platyhelminthes PRSs, (i) *Culex* densovirus through *Decapod penstyldensovirus 1*, (ii) *Parvovirinae* via chaphamaparvoviruses, (iii) *Aviadenovirus* and human betaherpesvirus 6 through *Chaphamaparvovirus-Parvovirinae*, and (iv) polyomaviruses via papillomaviruses may have evolved.

### Pairwise sequence similarity relationship of the parvo-NS1 DNA helicase domain with other DNA viruses.

**FIG 4:**
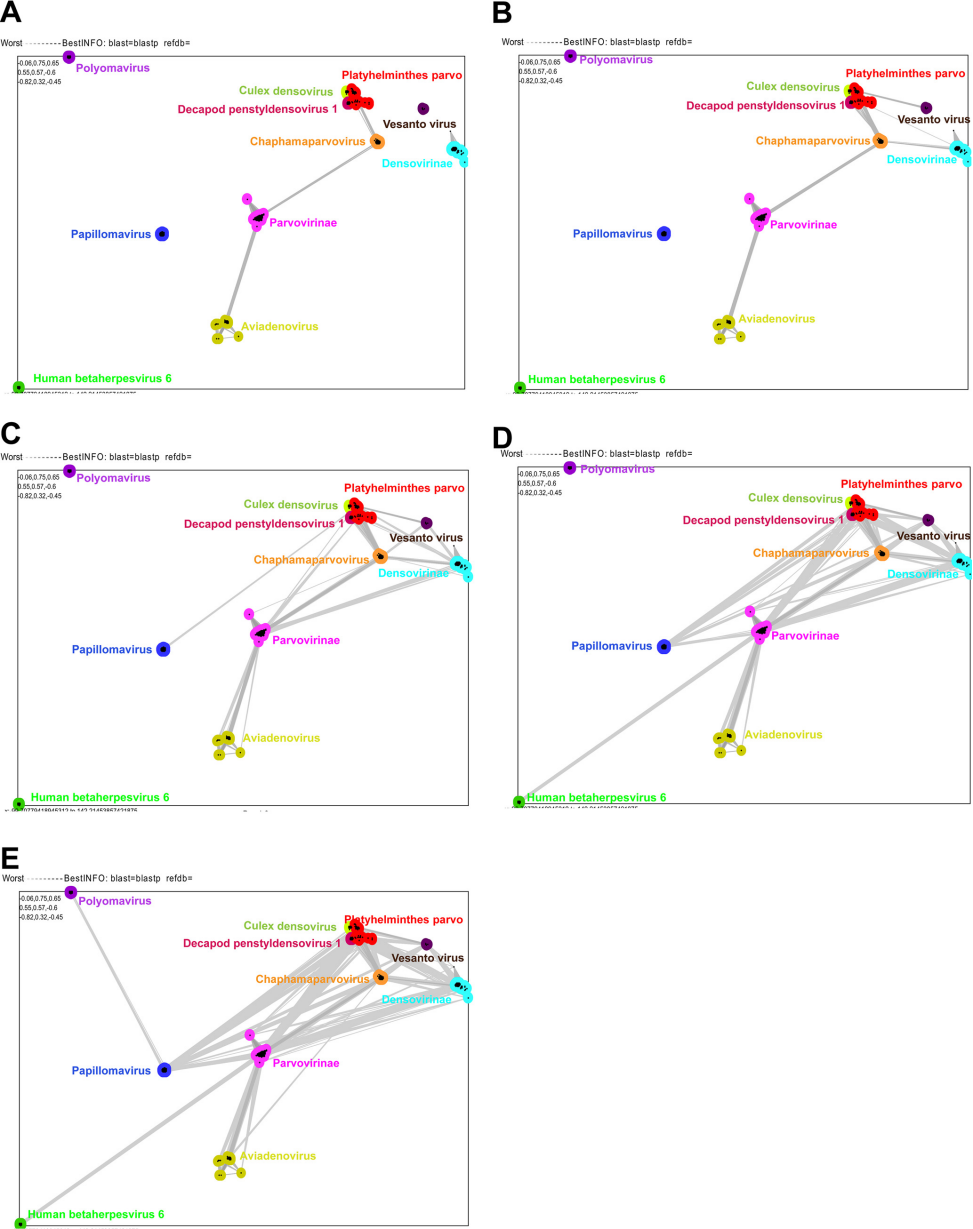
The CLANS pairwise similarity relationship of parvo-NS1 domain with other DNA viruses. CLANS (pairwise similarity network) analysis was carried out using a total of 2,938 amino acid sequences, such as the parvo-NS1 domains of parvovirus and related proteins in other DNA viruses (the details of strains that belong to different clusters are presented in Data Set S5 in the supplemental material). (A) The *P* value threshold of 1e–20 in CLANS is used to show the lines connecting the sequences. (B) The *P* value threshold of 1.2e–19 in CLANS is used to indicate the lines connecting the sequences. (C) The *P* value threshold of 1.2e–16 in CLANS is used to indicate the lines connecting the sequences. (D) The *P* value threshold of 1.2e–12 in CLANS is used to indicate the lines connecting the sequences. (E) The *P* value threshold of 1.2e–10in CLANS is used to indicate the lines connecting the sequences.

**FIG 5:**
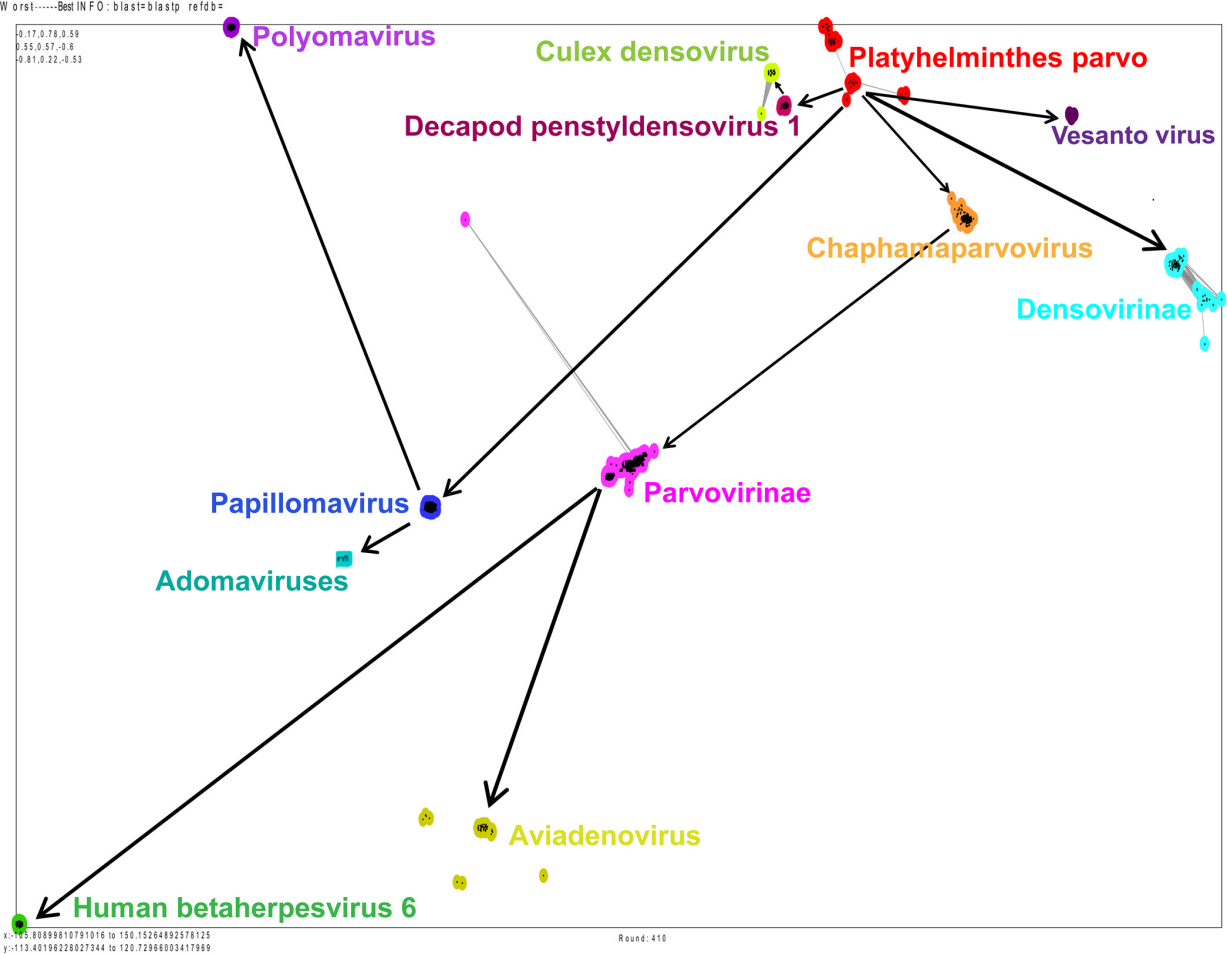
Proposed schematic diagram for the evolution of the DAN helicase domain in DNA viruses.

The whole family of conserved parvo-NS1 DNA helicase domain proteins is recommended to classify the *Parvoviridae* family of invertebrate- and vertebrate-infecting viruses (1). Further, homologous sequences of the parvo-NS1 DNA helicase domain are present in papillomavirus E1, polyomavirus large T-antigen, *Aviadenovirus* ORF2, and betaherpesvirus U94/Rep protein (15–18), so we performed CLANS analysis using this domain. At a *P* value threshold of 1e−35, Culex densovirus, *Decapod penstyldensovirus 1*, Platyhelminthes PRSs, *Densovirinae* viruses, *Chaphamaparvovirus*, *Parvovirinae*, papillomaviruses, polyomaviruses, *Aviadenovirus*, and betaherpesvirus formed independent orphan clusters without mutual evolutionary connection (Fig. S2D) (the details of strains that belong to different clusters are presented in Data Set S5 and S5a in the supplemental material). At a *P* value threshold of 1e−33, Platyhelminthes PRSs displayed an evolutionary link with *Decapod penstyldensovirus 1* (Fig. S2E). At a *P* value threshold of 1e−25, Platyhelminthes parvoviruses showed an evolutionary link with *Culex* densovirus through *Decapod penstyldensovirus 1* (Fig. S2F). Interestingly, at a *P* value threshold of 1e−20, Platyhelminthes PRSs displayed an evolutionary link with *Chaphamaparvovirus*, then *Chaphamaparvovirus* extended the evolutionary connection to *Parvovirinae*, and then *Parvovirinae* spread the evolutionary link to *Aviadenovirus* (Fig. 4A). At a *P* value threshold of 1.2e−19, *Densovirinae* showed an evolutionary association with Platyhelminthes PRSs and *Chaphamaparvovirus* (Fig. 4B). Next, at a *P* value threshold of 1.2e−16, Platyhelminthes PRSs established a direct evolutionary link with papillomaviruses (Fig. 4C). Likewise, at a *P* value threshold of 1.2e−12, *Parvovirinae* extended the evolutionary link to human betaherpesvirus 6 (Fig. 4D). Finally, at a *P* value threshold of 1.2e−10, papillomaviruses established a direct evolutionary link with polyomavirus (Fig. 4E). Furthermore, analysis using adomaviruses revealed that adomaviruses are closely related to papillomaviruses (see Fig. S3A to C in the supplemental material). After this, since DNA helicase/SF3 motif is present in the parvo-NS1 domain amino acid sequences used in this present study, we are interested to find out what result will be obtained if the sequences used in the previous study (22) to detect the evolutionary development of SF3 domain in other viruses (circular replication [Rep]-encoding single-stranded (CRESS) DNA virus, *Circoviridae*, genomoviruses, geminiviruses, *Nanoviridae*/*Alphasatellitidae*, smacovirus, plasmid-CRESS [pCRESS] sequences) and plasmids are used as an outgroup in CLANS analysis. In this analysis, the parvo-NS1 domain amino acid sequences used in the present study revealed an evolutionary link with the SF3 domain containing other viruses and plasmids used in previous studies at a *P* value threshold of 1e−5 (see Fig. S4A in the supplemental material) (the details of strains and sequences used in this analysis are presented in Data Set S5b). Also, at a *P* value threshold of 1e−8, an evolutionary link was revealed between the parvo-NS1 domain amino acid sequences used in the present study, whereas the evolutionary link between the sequences used in the present study and the outgroup (the SF3 domain in other viruses and plasmids used in the previous study) disappeared (Fig. S4B). This suggests that using or not using outgroup sequences does not change the evolution detected in the present study using CLANS analysis. Collectively, DNA helicase-level CLANS analysis revealed that Platyhelminthes PRSs might be the common ancestor of these viruses (Fig. 5). It has been suggested that the parvo-NS1 domain of *Chaphamaparvovirus* may have evolved first from Platyhelminthes PRSs, then *Parvovirinae* from *Chaphamaparvovirus*, and finally the parvo-NS1 domain in the *Aviadenovirus* and human betaherpesvirus 6 might have obtained the parvo-NS1 homologous domain via gene capture from a dependovirus (*Parvovirinae*) (Fig. 5). It can also be speculated that the DNA helicase of papillomaviruses may have led to the evolutionary diversification of adomaviruses’ and polyomaviruses’ DNA helicase after the DNA helicase of papillomaviruses evolved from the Platyhelminthes PRSs-NS1 domain (Fig. 5).

### Virus-host coevolution.

**FIG 6:**
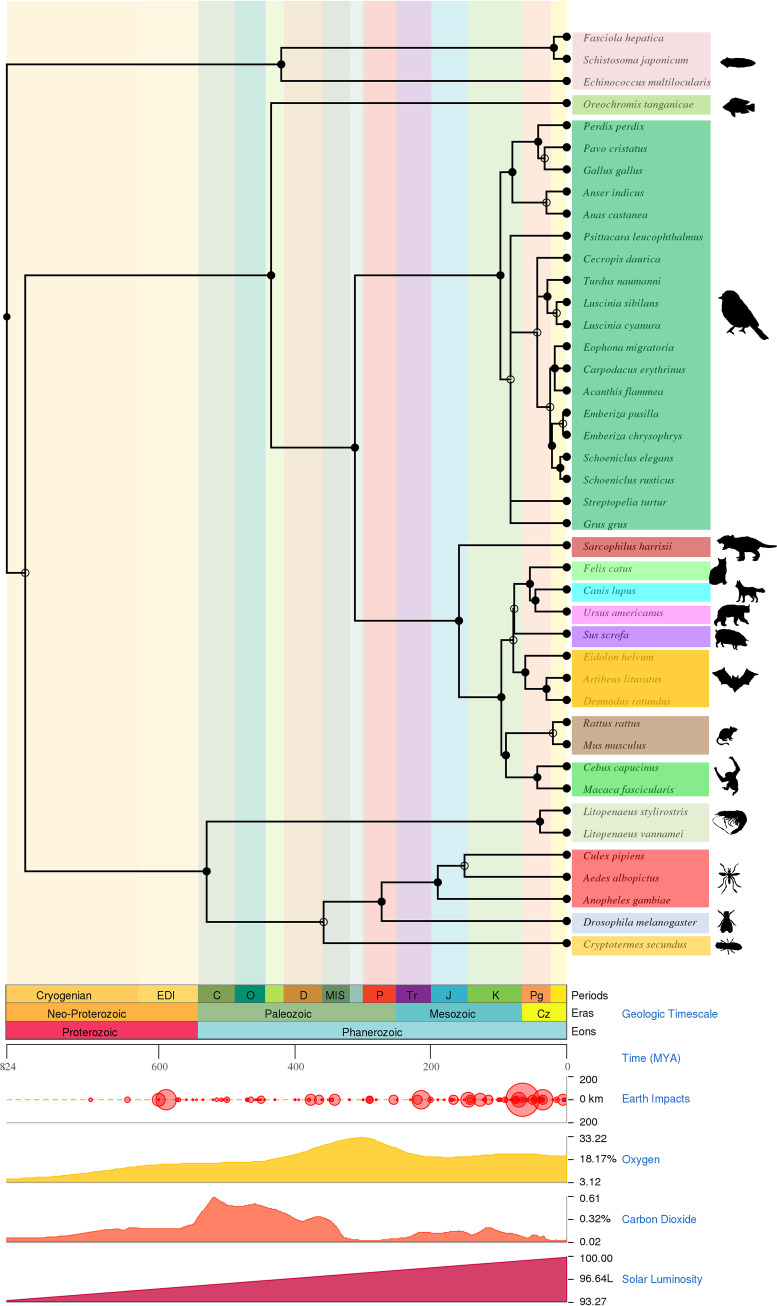
Timescale phylogenetic tree for hosts. The Timescale phylogenetic tree depicts the evolutionary origin and time of divergence for the host from platyhelminths to humans. The time is displayed in geological timescale (periods, eras, and eons), millions of years ago (MYA), Earth impact, oxygen, carbon dioxide, and solar luminosity measurements (details host and parvovirus sequences [*Hamaparvovirinae*] presented in Data Set S6 in the supplemental material).

**FIG 7:**
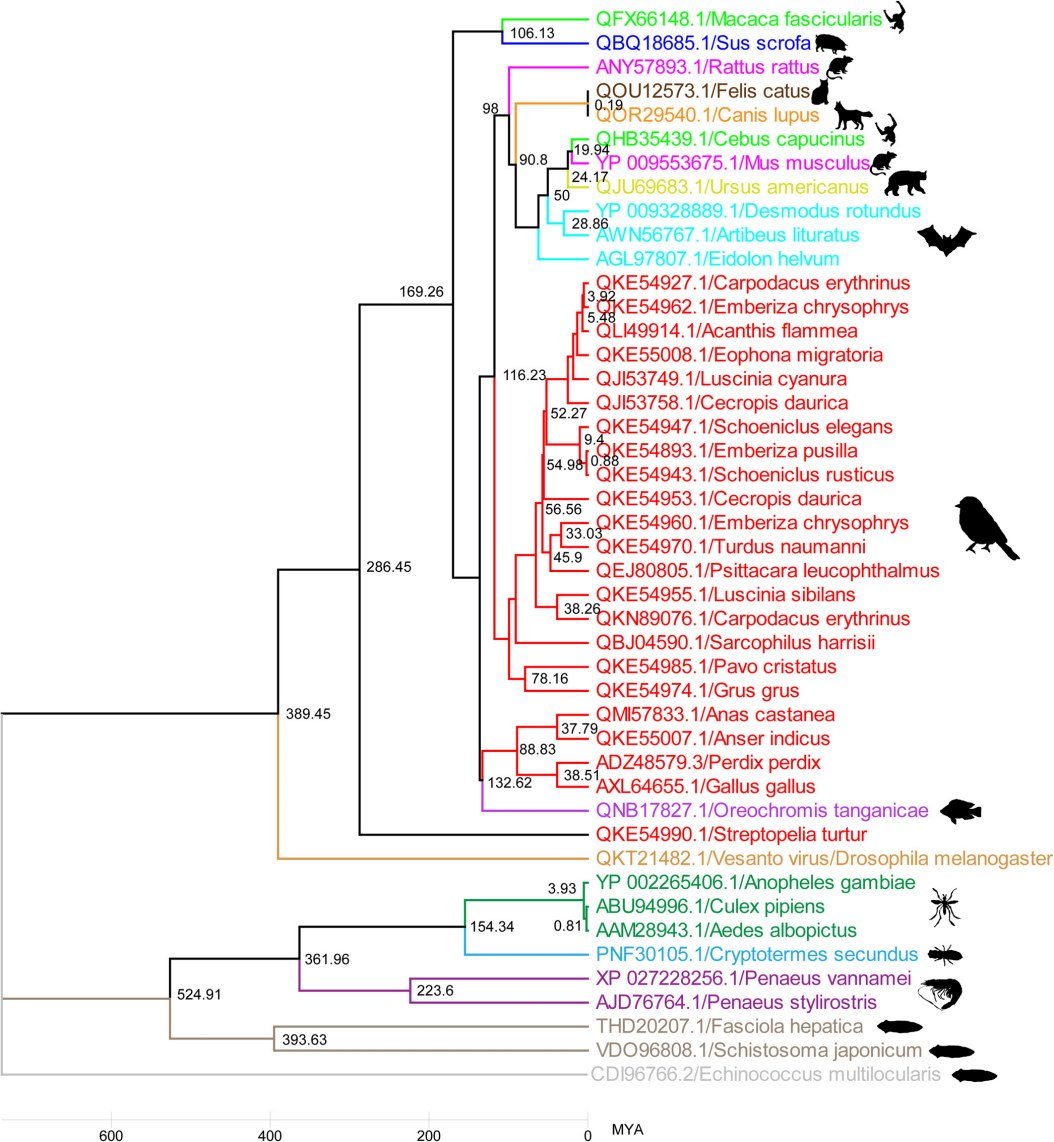
Relative timescale (RelTime) phylogenetic tree for parvo-NS1 domain. The relative timescale (RelTime) phylogenetic tree depicts the evolutionary origin and time of divergence for the parvovirus parvo-NS1 domains detected from platyhelminths to humans (the details of strains used in this analysis are presented in Data Set S7 in the supplemental material).

To further strengthen the evolutionary path of the DNA helicase of parvoviruses from the common ancestor Platyhelminthes PRSs, we performed separate TimeTree analyses for hosts and viruses followed by host-virus coevolution analysis (details host and parvovirus sequences [*Hamaparvovirinae*] presented in the Data Set S6 in the supplemental material). These analyses revealed that >800 million years ago (MYA), flatworms separated from arthropods, and then approximately 800 MYA, arthropods and fish evolved separately (Fig. 6). Later, in the arthropods, the evolutionary split between Malacostraca (Decapoda) and Insecta was revealed to be >500 MYA in the TimeTree (Fig. 6). TimeTree analysis indicated that approximately 450 MYA, fish separated from birds and mammals, and about 300 MYA, birds and mammals evolved apart (Fig. 6). In our TimeTree analyses, it appears that most of the invertebrate-infecting viruses separated from vertebrate-infecting viruses >500 MYA (Fig. 7). Further, it seems that the parvoviruses that infect invertebrates first separated from the viruses that infect fish around 132 MYA (Fig. 7) (the details of strains used in this analysis are presented in Data Set S7 in the supplemental material). TimeTree analysis revealed that the subsequent evolutionary division occurred between viruses affecting fish, birds, and mammals (Fig. 7). Finally, viruses that infect mammals have been shown to be separated from viruses that infect birds around 100 MYA (Fig. 7). Interestingly, the TimeTree for the evolution of hosts and the TimeTree for the evolution of viruses almost coincide.

**FIG 8:**
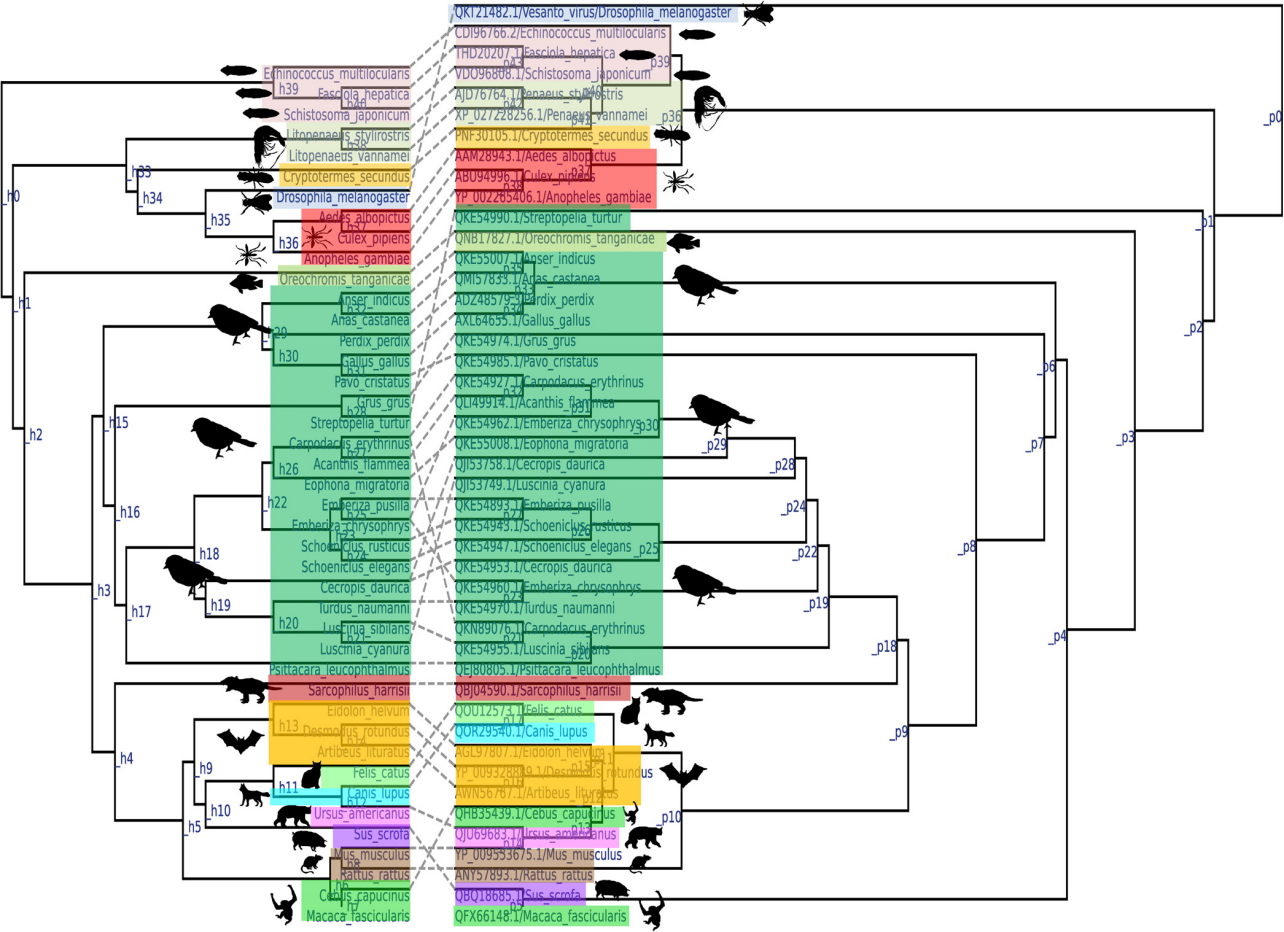
Virus-host coevolution tanglegram. The virus-host coevolution phylogenetic tree depicts the parvovirus parvo-NS1 domains detected from platyhelminths to humans. Virus-host coevolution analysis was performed using the eMPRess tool (the details of strains used in this analysis are presented in Data Set S8 in the supplemental material).

Finally, in virus-host coevolution analysis, viruses that infect Platyhelminthes may have appeared as early as Platyhelminthes. Later, when Malacostraca (Decapoda) evolved after Platyhelminthes, the viruses evolved with the host, and the viruses that infected Decapoda may have evolved after viruses that infect Platyhelminthes (Fig. 8; see also Fig. S5A to C in the supplemental material) (the details of strains used in this analysis are presented in Data Set S8 in the supplemental material). Next, as Insecta evolved after Decapoda, the viruses that infect them seem to have coevolved as well (Fig. 8; see also Fig. S5A to C). Similar virus-host coevolution was observed in fish, birds, and mammals (Fig. 8; see also Fig. S5A to C). Collectively, these analyses increased the possibility that the DNA helicase of Platyhelminthes PRSs was the common ancestor of the parvoviruses that coevolved with their hosts. Overall, these analyses increased the likelihood that DNA helicase of Platyhelminthes PRSs may be a common ancestor of parvoviruses and that parvoviruses may have evolved along with the evolution of their hosts.

## DISCUSSION

In the present study, the combination of phylogenetic trees, CLANS, and virus-host coevolution analysis based on the parvo-NS1 (DNA helicase) domain revealed that parvoviruses affecting vertebrates may have evolved from the common ancestor Platyhelminthes PRSs. Due to the lack of acceptable sequence alignment at the level of the complete parvo-NS1 protein, and the presence of the parvo-NS1 domain at the location of the alignment, it would be appropriate to determine the evolutionary path only through the results of parvo-NS1 domain-level phylogenetic and CLANS analyses. Also, it is worth noting that all viruses in the *Parvoviridae* family are related only at the Parvo-NS1 domain level, and this domain has been recommended to classify parvoviruses (1). Further, the present study shows that the evolutionary relationship of the parvo-NS1 domain in different families can be determined through phylogenetic analysis and the acceptable evolutionary path through CLANS analysis. Similarly, the evolutionary path of parvoviruses can be determined by the combination of CLANS and virus-host TimeTree-based coevolution analysis.

In the present study, TimeTree analysis for viruses and hosts and a virus-host coevolution tree analysis suggests that Platyhelminthes PRSs may be the first to appear. CLANS and virus-host coevolution analysis revealed that *Decapod penstyldensovirus 1* originated directly from the Platyhelminthes PRSs and then *Culex* densovirus from *Decapod penstyldensovirus 1*. Similarly, *Densovirinae* (infecting invertebrates) and *Chaphamaparvovirus* (infecting vertebrates) also appear to have evolved directly from Platyhelminthes PRSs. Subsequently, viruses belonging to the subfamily *Parvovirinae* that infect vertebrates have been shown to evolve from Platyhelminthes PRSs through *Chaphamaparvovirus.*

Conversely, the *Papillomaviridae* family is comprised of 5.7- to 8.6-kbp-long double-stranded DNA viruses that carry the early gene (three to six proteins [E1, E2, E4, E5, E6, and E7]) and the late gene (two capsid proteins [L2 and L1]) (6, 8). Of these, the viral proteins E1 and E2 are required to initiate viral DNA replication, where protein E1 contains the DNA helicase domain needed to initiate viral replication (6, 8, 13). *Papillomaviridae* viruses are known to infect vertebrates from fish to mammals (6, 8, 13). Similarly, *Polyomaviridae* viruses are 5-kbp-long circular dsDNA viruses that infect vertebrates from fish to mammals. They hold a large T-antigen (LTAg) DNA helicase domain to initiate virus replication (9). Further, our parvo-NS1 domain-based results revealed that papillomaviruses have a more distant evolutionary relationship with Platyhelminthes PRSs than *Decapod penstylhamaparvovirus 1*, *Densovirinae*, and *Chaphamaparvovirus*. *Decapod penstylhamaparvovirus 1* and *Chaphamaparvovirus*, which are closely related to Platyhelminthes PRSs, only have DNA helicase homologous sequences as well as between *Parvovirinae* and *Densovirinae* (1); they lack the VP protein PLA2 domains as those found in *Parvovirinae* and *Densovirinae* (1, 25). From these, chaphamaparvoviruses can be considered unique viruses with similarities to the DNA helicase of *Parvovirinae* and *Densovirinae*. Similarly, Platyhelminthes PRSs also have distinct viral sequences connecting *Decapod penstylhamaparvovirus 1*, *Densovirinae*, chaphamaparvoviruses, and papillomaviruses. Interestingly, our MAFFT alignment and BMGE curation-based phylogenetic and CLANS analyses indicated that *Parvovirinae* and papillomaviruses maintained almost identical evolutionary distances from Platyhelminthes PRSs. However, in the case of *Parvovirinae*, chaphamaparvoviruses form an evolutionary intermediate between the *Parvovirinae and* Platyhelminthes PRSs. In contrast, this type of evolutionary intermediate is missing between the papillomaviruses and Platyhelminthes PRSs. Furthermore, host-virus coevolution analysis using Platyhelminthes PRSs, *Decapod penstylhamaparvovirus 1*, *Culex* densovirus, and chaphamaparvoviruses have shown that when vertebrates evolve from invertebrates, chaphamaparvoviruses also evolve from invertebrate-infecting viruses along with their hosts. Therefore, it can be inferred that chaphamaparvoviruses (including *Ichthamaparvovirus*) were the first parvoviruses infecting vertebrates that directly evolved from viruses that infect invertebrates along with host evolution. Late in the evolution of parvoviruses, it can be speculated that a subfamily of *Parvovirinae* viruses emerged from the chaphamaparvoviruses. Thus, the following hypotheses can be inferred from the absence of intermediate evolutionary viruses between papillomaviruses and Platyhelminthes PRSs: (i) papillomaviruses that infect invertebrates remained undetected and may be intermediate viruses that evolved between papillomaviruses and Platyhelminthes PRSs, and (ii) at the time of the emergence of parvoviruses that infect vertebrates from Platyhelminthes PRSs, the lineages of some viruses may have evolved into papillomaviruses that infect vertebrates. Considering the available data, it has been suggested that the DNA helicase of papillomaviruses may have evolved directly from the Platyhelminthes PRSs in the early stages of the evolution of parvoviruses and that the DNA helicase of papillomaviruses led to the evolution of adomaviruses and polyomaviruses that infect vertebrates.

Furthermore, our CLANS analysis also supported the evolution of the aviadenovirus ORF2 and U94/Rep of betaherpesviruses from the *Parvovirinae* subfamily. Next, our in-depth NCBI BLAST analysis of aviadenovirus ORF2 and betaherpesvirus U94/Rep revealed that these sequences are closely related to a dependovirus (adeno-associated virus) belonging to the *Parvovirinae* subfamily. Moreover, the *Adenoviridae* family consists of linear double-stranded DNA viruses ranging from 25 to 48 kbp long with five genes and contains many functional proteins (26, 27). Only the *Aviadenovirus* genus out of the five genera in the *Adenoviridae* family encodes ORF2, a homolog of parvo-NS1 (19). Similarly, the *Herpesviridae* family comprises viruses with a linear double-stranded DNA ranging from 124 to 259 kbp long and encoding 70 to 200 proteins (28, 29). Curiously, only HHV-6, RCMV, and MsHV, which belong to the subfamily betaherpesvirinae, encode U94/Rep, which is homologous to parvo-NS1 (15–18). From these, it can be realized that the parvo-NS1 domain-containing proteins are not essentially required to replicate all adenovirus and betaherpesviruses. Remarkably, dependoviruses usually do not replicate independently in the host cells and always depend on a helper virus, such as an adenovirus or a herpesvirus, for successful viral replication (4, 5). All of this suggests that when adenoviruses or herpesviruses aid in the replication of a dependovirus, some adenoviruses (*Aviadenovirus*) and herpesviruses (human betaherpesvirus 6) may have obtained the parvo-NS1 homologous domain via gene capture from a dependovirus at the later stage of parvovirus evolution.

Although DNA helicase is a critical domain for the replication of DNA viruses, successful replication of viruses in different DNA virus families requires interaction/interference of host proteins with multiple cellular types of machinery/pathways and different nick formation processes (30). Also, looking at the available data, it appears that the parvo-NS1 domain evolved with Platyhelminthes PRSs as a common ancestor. Whereas, the hypotheses that this domain may have originated through ancient co-infection of the same cell, recombination, gene capture, and horizontal and vertical gene transfer cannot be ignored entirely.

In conclusion, to the best of our knowledge, this is the first report on the origin and evolutionary path of DNA viruses (DNA helicase domain) that infect vertebrates from the common ancestor of invertebrates (Platyhelminthes PRSs). DNA helicase domain of papillomaviruses, adomaviruses, and polyomavirus potentially evolved from the Platyhelminthes PRSs in the early stages of the evolution of parvoviruses. Then, the *Parvovirinae* viruses' DNA helicase domain evolved from the Platyhelminthes PRSs through *Chaphamaparvovirus*. Finally, *Aviadenovirus* and human betaherpesvirus 6 may have evolved with DNA helicase via gene capture from dependoviruses at the later stage of parvovirus evolution. These findings shed light on the evolutionary path, origin, common ancestor, and immediate ancestor of DNA viruses.

## MATERIALS AND METHODS

### Data collection, sequence searches, and data curation.

We retrieved approximately 3,000 to 4,000 sequences of parvo-NS1 complete protein and the amino acid sequences of the Parvo-NS1 domain (DNA helicase domain) from invertebrate parvovirus-related sequences (PRSs), parvoviruses, papillomaviruses, polyomaviruses, herpesviruses, and aviadenoviruses from the NCBI database (https://www.ncbi.nlm.nih.gov/protein/). We subjected each virus to the above different groups for NCBI BLAST analysis. We retrieved the amino acid sequences of the various viruses associated with each group from the NCBI public database (https://blast.ncbi.nlm.nih.gov/Blast.cgi?PAGE=Proteins). We have performed the NCBI BLAST analysis using the protein-protein BLAST algorithm in BLASTP with the following parameters: the nonredundant protein sequences (nr) database, except threshold 0.05, word size 6, max matches in a query range 0, BLOSUM62 matrix, gap costs-existence: 11 extension: 1, conditional compositional score matrix adjustments for compositional adjustments. We also verified the parvo-NS1 domains in the retrieved sequences using the Conserved Domain search tool (https://www.ncbi.nlm.nih.gov/Structure/cdd/wrpsb.cgi?). The search for parvo-NS1 domains was carried out in the Conserved Domain search tool using the following parameters: CDD v3.19-58235 PSSms-database; expect value threshold, 0.01; composition-based statistics adjustment-applied, performed by the maximum number of hits-500 (31). Furthermore, the parvo-NS1 domains in other DNA viruses have been identified using the conserved domain architecture retrieval tool (https://www.ncbi.nlm.nih.gov/Structure/lexington/lexington.cgi). To search the parvo-NS1 domain for other DNA viruses, we used the NCBI Virus taxonomy tree code INCLSPAN[10239] and the parvo-NS1 domain code cl24009[parvo_NS1] in the conserved domain architecture retrieval tool (32).

### Phylogenetic analyses. (i) Using phylogenetic analysis.

The retrieved sequences (parvo-NS1 complete protein or parvo-NS1 domain amino acid sequences) were first aligned in MAFFT 7.407_1 using a gap extend penalty of 0.123 and a gap opening penalty of 1.53 (33, 34). FastTree maximum-likelihood phylogenetic trees were performed in Galaxy version 2.1.11+galaxy1 (35–37), with the following evolutionary model: LG, gamma distribution, amino acid distances-BLOSUM45 Joins-balanced Support-SH-like 1000, and search of Normal +NNI +SPR +ML-NNI. Finally, the phylogenetic tree was visualized using the Interactive Tree of Life (iTOL) v5 (38).

The retrieved parvo-NS1 domain amino acid sequences from different DNA viruses were first aligned in MAFFT 7.407_1 using a gap extend penalty of 0.123 and a gap opening penalty of 1.53 (33, 34). Sequences were aligned in MAFFT and then in BMGE 1.12_1 with the following parameters to trim the ambiguously aligned areas and improve the accuracy of the phylogenetic relationship: estimated matrix, BLOSUM62; sliding windows size, 3; maximum entropy threshold, 0.5; gap rate cutoff [0–1], 0.5; and minimum block size, 5 (20, 33). This was followed by phylogenetic tree construction in FastTree 2.1.11 (35–37), with the following evolutionary model: LG, gamma distribution, amino acid distances-BLOSUM45 Joins-balanced Support-SH-like 1000, and search of Normal +NNI +SPR +ML-NNI. Finally, the phylogenetic tree was visualized using the Interactive Tree of Life (iTOL) v5 (38). The rooted tree and unrooted trees were created using the Interactive Tree of Life (iTOL) v5 (38).

### Timescale phylogenetic tree. (a) Timescale phylogenetic tree for hosts.

The time scale phylogenetic tree for hosts was created using TimeTree: The Timescale of Life (http://www.timetree.org/search/goto_timetree) (39). A group of species or a custom list of hosts was used to build the timescale phylogenetic tree for hosts.

### (ii) Relative timescale (RelTime) phylogenetic tree for viruses.

The phylogenetic tree was first created in MEGA X software using the maximum likelihood method and the JTT matrix-based model with neighbor-joining and BioNJ algorithms (40) to construct the relative timescale (RelTime) phylogenetic tree for the viral parvo-NS1 domains (41). The resulting phylogenetic tree was exported in the Newick format. The phylogenetic tree in the Newick format and the source MEGA-aligned sequences were used in MEGA X’s RelTime-ML to create a relative timescale (RelTime) phylogenetic tree for the viral parvo-NS1 domains. Finally, the relative timescale (RelTime) phylogenetic tree was inferred using the RelTime method (42, 43). After this, the RelTime phylogenetic tree was calibrated with the time specified in previous studies (6, 44–46).

### Virus-host coevolution analysis.

Virus-host coevolution analysis was performed using the eMPRess tool (47–50). A Newick format phylogenetic tree for hosts, a Newick format phylogenetic tree for viruses, and a tip mapping file for virus-hosts are needed to perform coevolution analysis in eMPRess. First, the Timescale phylogenetic tree for hosts was created using FaceTimeTree: The Timescale of Life (http://www.timetree.org/search/goto_timetree) (39). A group of species or a custom list of hosts was used to build the timescale phylogenetic tree for hosts. The resulting phylogenetic tree was exported in Newick format and stored. Next, the phylogenetic tree for the parvo-NS1 domains of viruses was created in MEGA7 using the evolutionary distances in the unweighted pair group method using average linkages (UPGMA) method using the equal input, gamma distribution (shape parameter = 5), and by removing all ambiguous positions for each sequence pair. The resulting phylogenetic tree was exported in Newick format and then stored. After this, the tip mapping files for each virus-host were created in the tip mapping format. The virus-host coevolution tanglegram was first generated in eMPRess using the Newick format phylogenetic tree for hosts, the phylogenetic tree in the Newick format for viruses, and the tip mapping files for virus hosts. Then, we looked at the event cost regions for coevolution. Next, two clusters were calculated to compute the reconciliations, and a virus-host coevolution tree was created for each cluster. The *P* value histogram for virus-host coevolution was then measured.

### CLANS analysis.

CLANS analysis was performed in the Toolkit (https://toolkit.tuebingen.mpg.de/tools/clans) (51–53). The retrieved parvo-NS1 complete protein or parvo-NS1 domain amino acid sequences were first converted into the FASTA format. FASTA-formatted sequences were subjected to a pairwise sequence similarity calculation using the CLANS tool in the Toolkit. BLOSUM62 scoring matrix and BLAST HSP (high scoring pair) up to a *P* value of 1E−2 for the CLANS analyses. The files obtained from the CLANS analysis were then visualized using the clans.jar tool in Java (21). The clans.jar tool uses at least 50,000 to 100,000 rounds to display links and clusters.

### Data availability.

We have retrieved the nucleotide sequences from publicly available NCBI databases. Further, all the nucleotide sequence accession numbers and names are indicated in the respective figures and supplemental data.
