# Supplementary material for: Deciphering the Origin of DNA Viruses (Replication-Associated Parvo-NS1) That Infect Vertebrates from Invertebrate-Infecting Viruses
Source: Microbiol Spectr. 2023 Jun 22;11(4):e04570-22. doi: 10.1128/spectrum.04570-22 (PMC10433990; doi:10.1128/spectrum.04570-22)
Supplement: Supplemental file 6 — Figures S1 to S5. Download spectrum.04570-22-s0001.pdf, PDF file, 1.5 MB [file spectrum.04570-22-s0001.pdf]

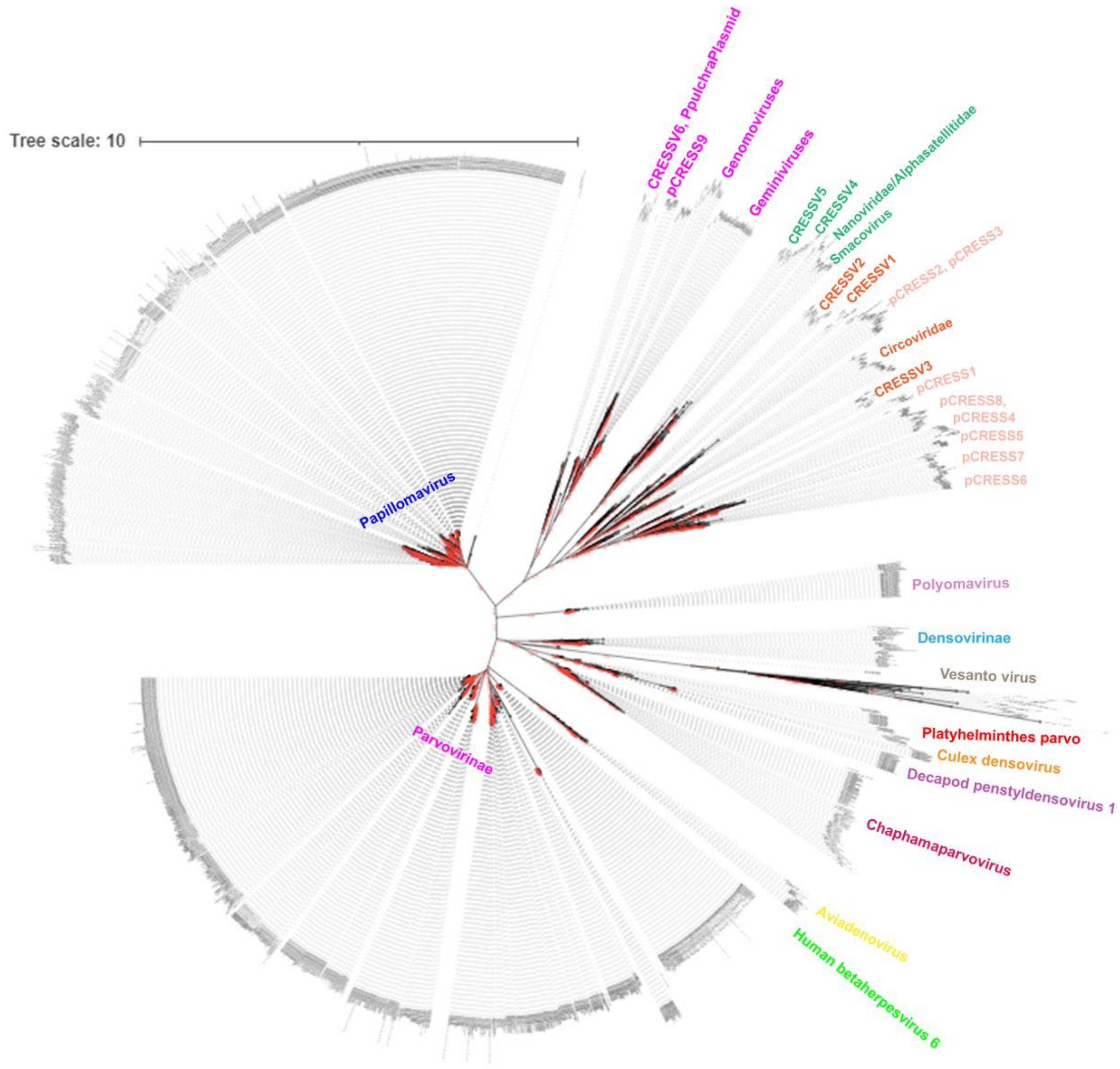

Supplementary Figure 1

Supplementary Figures 1: Phylogenetic relationship of Parvo-NS1 domains with other DNA viruses and plasmids. A total of 3647 amino acid sequences were used, the details of strains that belong to different viruses are presented in **Supplementary Data 2a**.

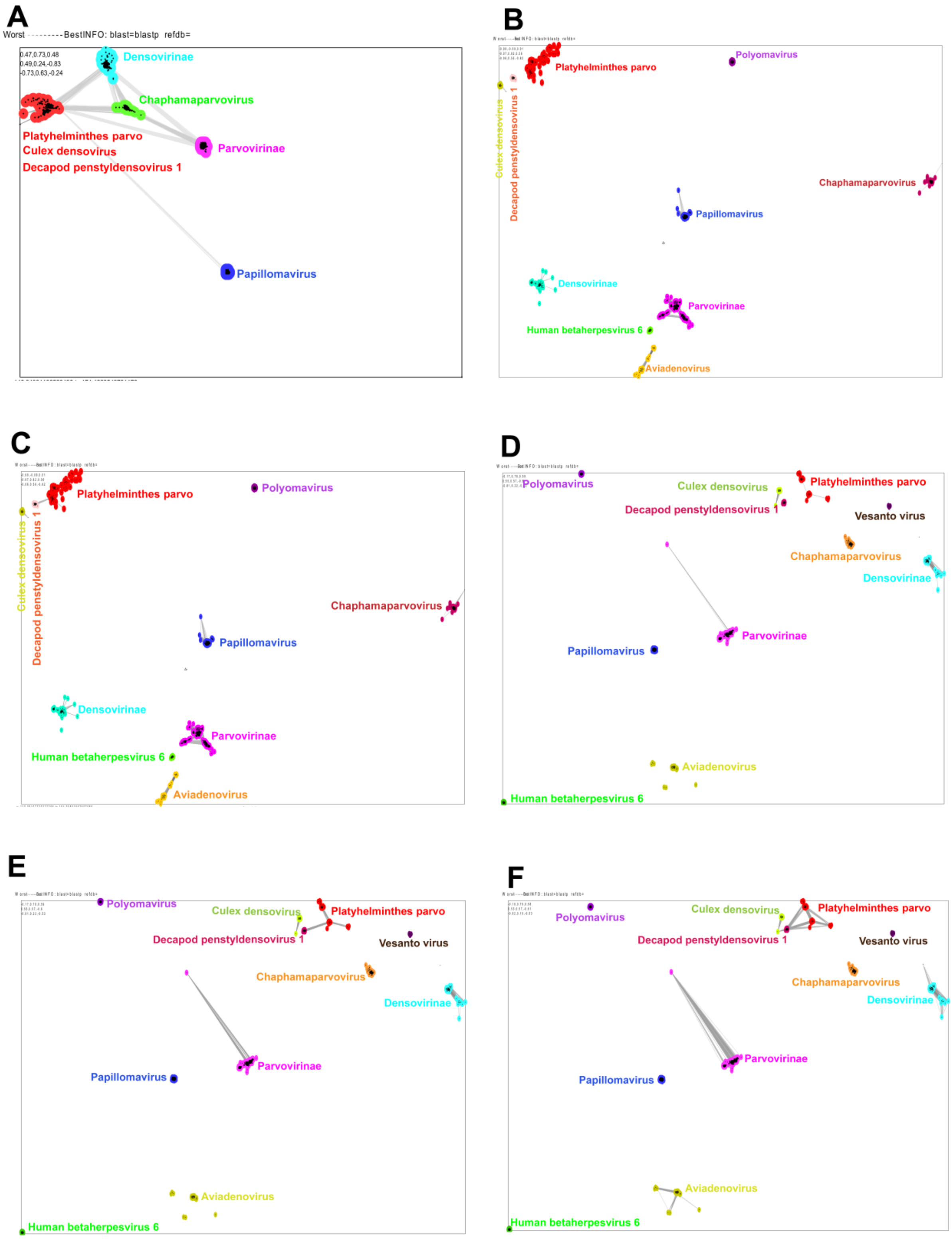

Supplementary Figure 2

Supplementary Figures 2: The CLANS pairwise similarity relationship of complete Parvo-NS1 and Parvo-NS1 domain with other DNA viruses. (A) . CLANS analysis was carried out using a total of 4362 amino acid sequences (amino acid sequences details presented in **Supplementary Data 3**), such as the entire length of the protein sequence of

Parvo-NS1 and its relative papillomavirus E1. At the p-value threshold of 1.0e-13, the parvovirus family formed four distinct clusters: (i) Platyhelminthes PRSs, Culex densovirus, and *Decapod penstyldensovirus 1*; (ii) *Densovirinae*, (iii) *Chaphamaparvovirus*, and (iv) *Parvovirinae*. The papillomavirus E1 displayed a direct evolutionary connection with the cluster formed by Platyhelminthes PRSs, Culex densovirus, and *Decapod penstyldensovirus 1*. (**B**) CLANS analysis was carried out using a total of 3118 amino acid sequences, such as the complete protein of parvovirus parvo-NS1 and related proteins in other DNA viruses. (The details of strains that belong to different clusters are presented in **supplementary data 4**). A p-value threshold of 1e-48 formed independent orphan clusters without mutual evolutionary contact. (**C**) A p-value threshold of 1e-44, Platyhelminthes PRSs showed an evolutionary link with *Decapod penstyldensovirus 1*. CLANS analysis was carried out using a total of 3118 amino acid sequences, such as the complete protein of parvovirus parvo-NS1 and related proteins in other DNA viruses. (The details of strains that belong to different clusters are presented in **supplementary data 4**). (**D**) CLANS analysis was carried out using a total of 2938 amino acid sequences, such as the parvo-NS1 domains of parvovirus and related proteins in other DNA viruses (The details of strains that belong to different clusters are presented in **supplementary data 5**). A p-value threshold of 1e-35 formed independent orphan clusters without mutual evolutionary contact. (**E**) At a p-value threshold of 1e-33, Platyhelminthes PRSs displayed an evolutionary link with *Decapod penstyldensovirus 1*. CLANS analysis was carried out using a total of 2938 amino acid sequences, such as the parvo-NS1 domains of parvovirus and related proteins in other DNA viruses. (**F**) At a p-value threshold of 1e-25, Platyhelminthes parvoviruses showed an evolutionary link with Culex densovirus through *Decapod penstyldensovirus 1*. CLANS analysis was carried out using a total of 2938 amino acid sequences, such as the parvo-NS1 domains of parvovirus and related proteins in other DNA viruses (The details of strains that belong to different clusters are presented in **supplementary data 5**).

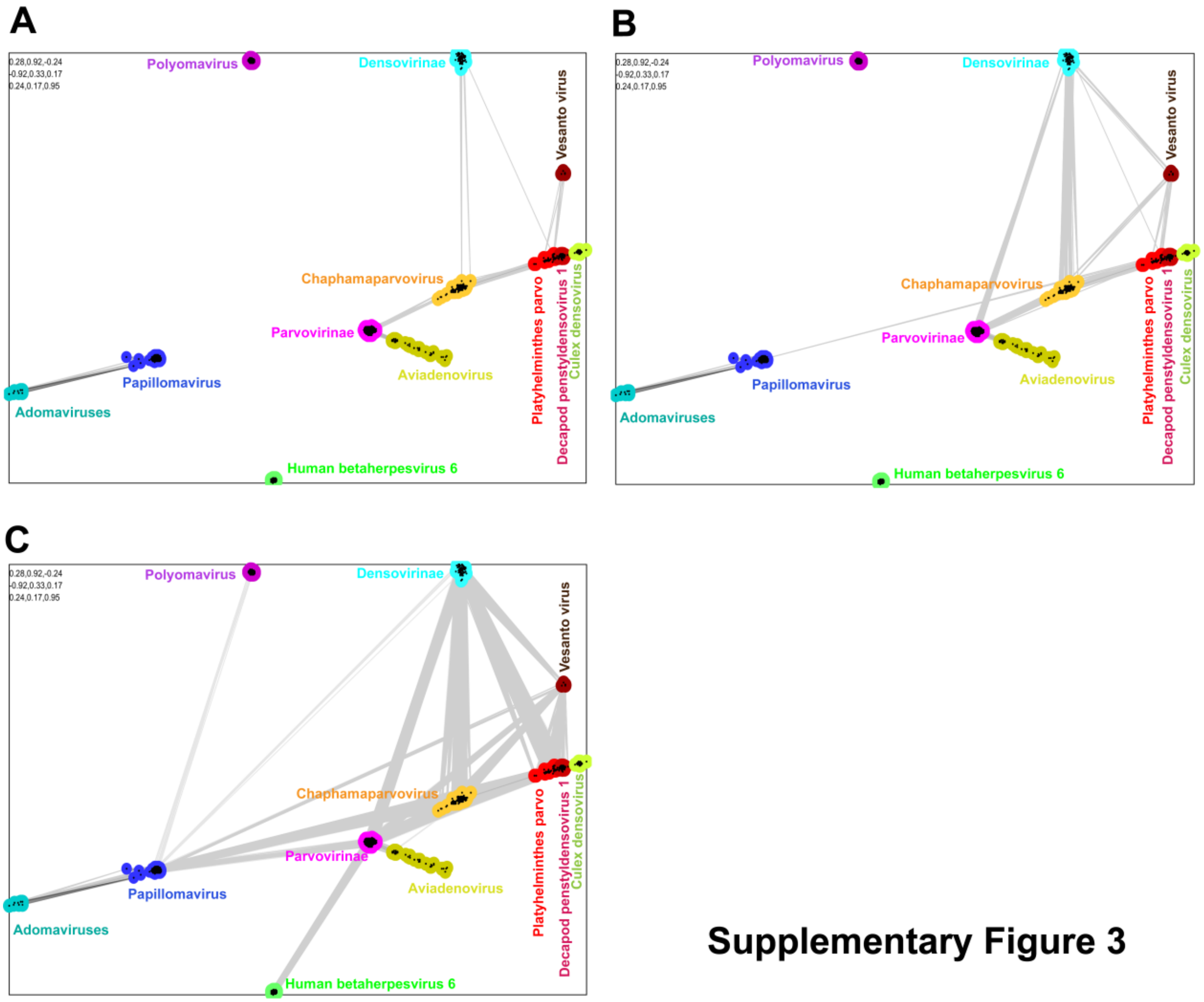

Supplementary Figure 3

Supplementary Figures 3: The CLANS pairwise similarity relationship of Parvo-NS1 domain with Papillomaviruses, adomaviruses, and **other DNA viruses.** (**A**) At a p-value threshold of 1e-19, Adomaviruses displayed an evolutionary link with Papillomaviruses. Papillomaviruses extended the evolutionary link to Platyhelminthes PRSs At a p-value threshold of 1e-17 (**B**); and 1e-10 (**C**). (The details of strains that belong to different clusters are presented in **supplementary data 9**)

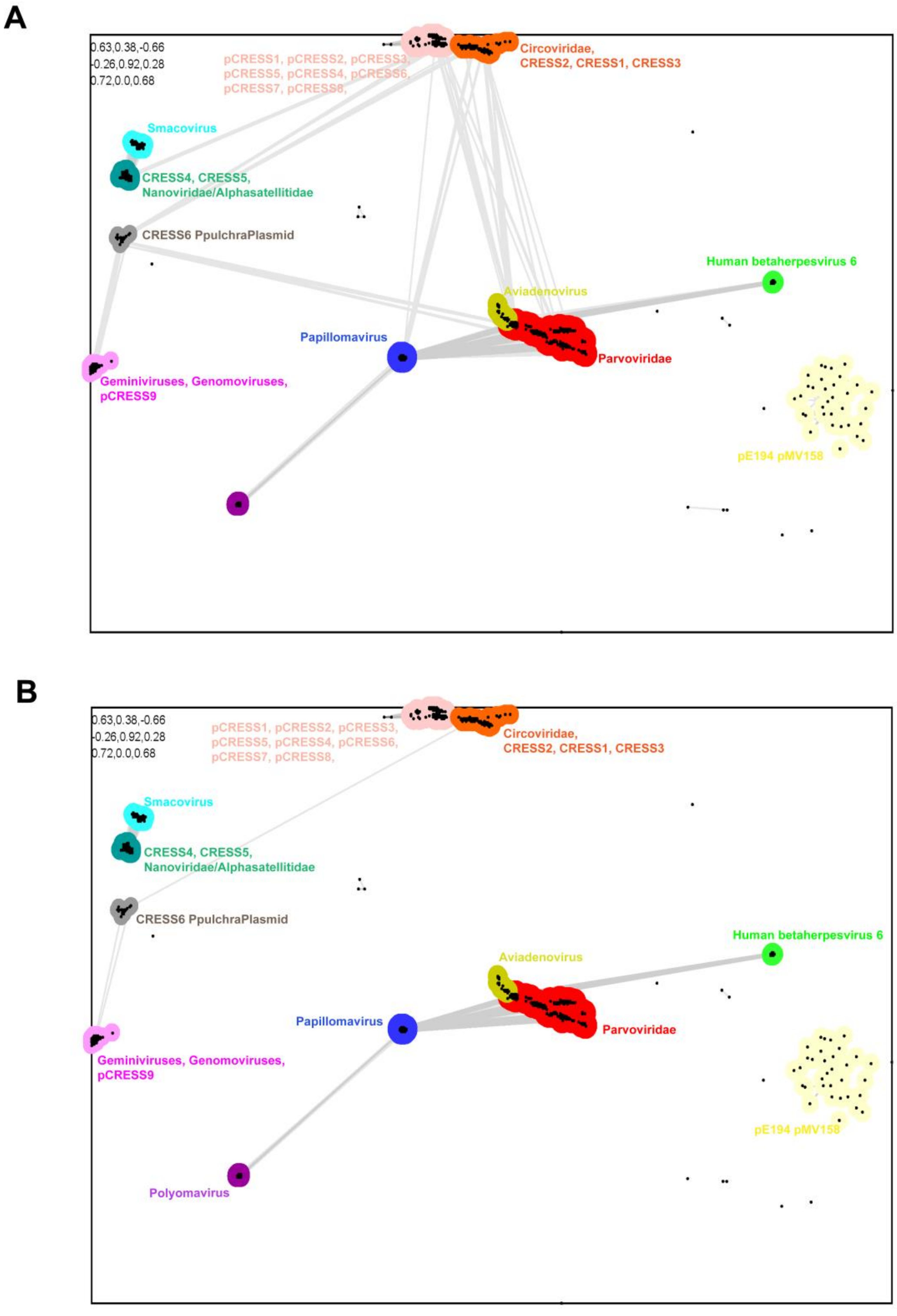

Supplementary Figure 4

Supplementary Figures 4: The CLANS pairwise similarity relationship of Parvo-NS1 domain with other DNA viruses and plasmids. The details of strains and sequences used in this analysis are presented in **supplementary data 5b**. (**A**) The p-value threshold of 1e-5 in CLANS is used to show the lines connecting the sequences. (**B**) The p-value threshold of 1e-8 in CLANS is used to indicate the lines connecting the sequences.

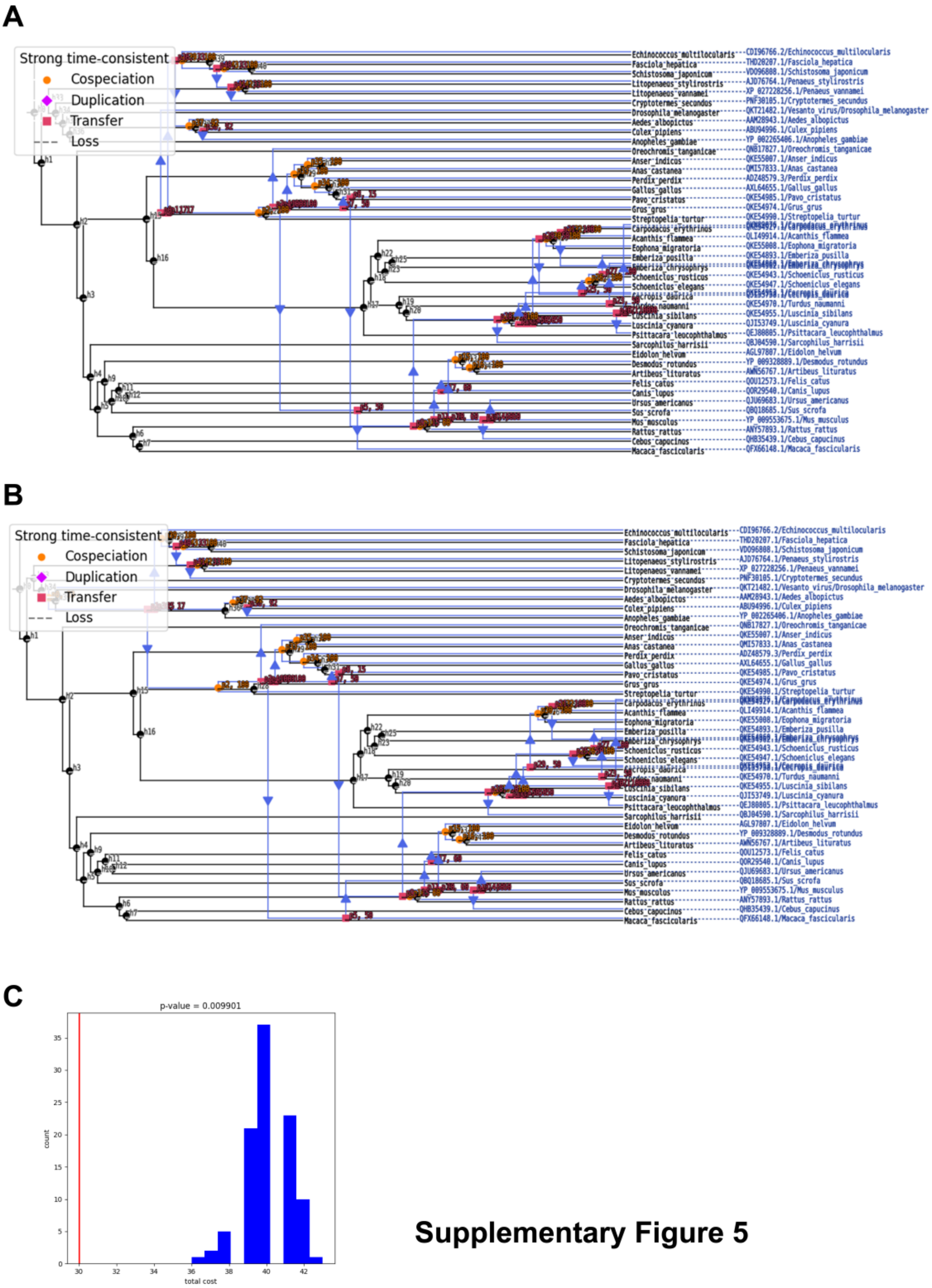

Supplementary Figures 5: Parvovirus-host co-evolution. (**A-B**) Illustration of parvovirus-host co-evolution using the Parvo-NS1 domains found from Plattihelminths to humans. The parvovirus-host co-evolution tree was created using the eMPRess tool. Two clusters were calculated to compute the reconciliations, and a virus-host co-evolution tree was created for each cluster. (A) Represent the cluster 1 based parvovirus-host co-evolution tree, and (**B**) Represent the cluster 2 based parvovirus-host co-evolution tree. (**C**) Displaying the p-value for the parvovirus-host co-evolution tree in the eMPRess tool.
